# Feasibility of a Remotely Delivered Strength and Balance Training Program for Older Adults with Cancer

**DOI:** 10.3390/curroncol28060374

**Published:** 2021-11-02

**Authors:** Schroder Sattar, Kristen Haase, Kelly Penz, Corrie Effa, Joni Nedeljak, Haji Chalchal, Osama Souied, Eitan Amir, Eric Pitters, Diane Campbell, Shabbir Alibhai, Margaret L. McNeely

**Affiliations:** 1 College of Nursing, University of Saskatchewan, Regina, SK S4T 0H8, Canada; kelly.penz@usask.ca; 2 Faculty of Applied Science, School of Nursing, University of British Columbia, Vancouver, BC V6T 2B5, Canada; kristen.haase@ubc.ca; 3 Faculty of Rehabilitation Medicine, University of Alberta, Edmonton, AB T6G 2G4, Canada; ceffa@ualberta.ca (C.E.); jnedelja@ualberta.ca (J.N.); mmcneely@ualberta.ca (M.L.M.); 4 Department of Medicine, University of Saskatchewan, Saskatoon, SK S7N 0X8, Canada; haji.chalchal@saskcancer.ca (H.C.); osama.souied@saskcancer.ca (O.S.); 5 Department of Medical Oncology, University Health Network, Toronto, ON M5G 2C1, Canada; eitan.amir@uhn.ca; 6 Patient Representative, Ajax, ON L1S 5C2, Canada; ericpitters@rogers.com; 7 College of Nursing, University of Saskatchewan, Saskatoon, SK S7N 5E5, Canada; diane.campbell@usask.ca; 8 Department of Medicine, University of Toronto, Toronto, ON M5S 1A8, Canada; shabbier.alibhai@uhn.ca; 9 Institute of Health Policy, Management, and Evaluation, University of Toronto, Toronto, ON M5T 3M6, Canada; 10 General Internal Medicine, University Health Network, Toronto, ON M5G 2C4, Canada; 11 Department of Oncology, Cross Cancer Institute, Edmonton, AB T6G 1Z2, Canada

**Keywords:** falls, resistance training, balance training, exercise intervention, physical performance measures

## Abstract

Falls are a major issue among older adults with cancer and lead to interruptions in cancer treatment. Resistance and balance training can prevent falls in older adults, but minimal evidence is available regarding the older cancer population, who often have unique risk factors. We used a pre–post design to assess the feasibility of a remotely delivered exercise program that progressed in difficulty and its efficacy on lower body strength, balance, and falls in older adults with cancer who had prior in-person exercise experience. Twenty-six older adults with cancer completed the intervention. Attendance rate for the virtual component was 97.6% and for the independent component was 84.7%. Participants perceived the program as rewarding and enjoyable (100%), felt this program prepared them to exercise on their own (92%), were confident to continue exercising on their own (81%), and would recommend the program to other patients (100%). The median balance score at baseline and end-of-study was 4 (IQR = 0). The median chair-stand time decreased from 9.2 s (IQR = 3.13) to 7.7 s (IQR = 4.6). A statistically significant difference in lower body strength (*r* = 0.68, *p* = 0.001) was detected post-intervention. The findings from this study can inform the design of a larger randomized trial.

## 1. Introduction

Falls are a major concern in older adults with cancer due to the disease and effects of its treatments [1], which can increase the risk for falls and fall injuries [2,3,4]. This population has higher fall rates and fall injuries than their cancer-free counterparts [5,6], and one-quarter of injuries in older adults with cancer involve bone fractures [7]. Moreover, one in 20 falls leads to interruption in older patients’ treatment [7], which may result in adverse implications on disease trajectory and health outcomes. Levels of physical activity (PA) in the older cancer population often decline after diagnosis and during treatment [8] and do not return to pre-treatment levels without formal intervention [9,10]. Furthermore, advanced age is associated with greater sedentary behavior [11], and the tendency for less PA is exacerbated, as patients are often advised to reduce activities when feeling fatigued or experiencing treatment side effects [12,13].

Many falls are preventable [14]. Mounting evidence demonstrates that strength and balance training is effective in reducing fall rates in the general geriatric population [14,15]. However, evidence on effective interventions to reduce falls in the geriatric oncology setting is lacking [16]. Older adults with cancer may have unique risk factors for sarcopenia and falls [17,18]. In particular, acute effects of treatment such as chemotherapy-induced peripheral neuropathy (CIPN) may give rise to impairments that are often sudden [19]. Simply extrapolating results from the larger body of geriatric exercise trials is not sufficient to inform how exercise should be prescribed for geriatric oncology patients [8]. ‘Activity prescription’ is currently underutilized in clinical practice in oncology [8], and exercise intervention trials for older patients with cancer and cancer survivors are necessary to help inform the use of exercise to treat cancer and treatment-related toxicities including sarcopenia and falls [20]. Establishing the potential of exercise to improve strength and balance and reduce falls in older adults with cancer—even in a small sample—would create an important foundation for larger studies and the development of informed policies that can be adopted by cancer care organizations as standard clinical practice.

Finally, cancer is an age-associated disease [21], with individuals > 65 years accounting for 70% of all newly diagnosed cancers and at least 70% of all cancer deaths [21]. The number of older adults is expected to double in the next 20 years; correspondingly, the number of new cancer cases is expected to increase by 40% in the next 15 years, mostly among older adults [22]. Evidence consistently demonstrates that the COVID-19 pandemic has disproportionately impacted older adults, with mortality greater as age and comorbidities increase [23]. Patients with comorbidities, including cancer, are more vulnerable [24] and have been advised to minimize outings and observe physical distancing [24]. In light of the aging population, and the circumstances created by the pandemic, it is timely to initiate innovative exercise intervention programs for older adults with cancer, such as using virtual intervention strategies, to enable them to stay active within the safety of their own homes. The primary objective of this study was to investigate the feasibility of an 8-week, remotely delivered PA e-intervention. The secondary objective was to determine the preliminary effects of the program on lower body strength, balance, and falls in community-dwelling older adults with cancer.

## 2. Materials and Methods

This remotely delivered exercise program utilized a single-group, pre- and post-test design. Due to the COVID-19 pandemic, research activities such as patient recruitment at major comprehensive cancer cancers were not viable. Therefore, we recruited older adults receiving care at the Cancer Rehabilitation Clinic at the University of Alberta. Clinic staff assisted by providing information about the study, with potential participants then contacting the study staff by email or telephone. To ensure physical suitability to partake in the program, participants were also assessed by telephone with the Physical Activity Readiness Questionnaire (PAR-Q) screening form [25].

Eligible participants met the following criteria: patients aged 65 and over; with a confirmed diagnosis of lung, breast, prostate, or colorectal cancer; who were receiving active cancer treatment or had completed cancer treatment within the past 6 years. Exclusion criteria were as follows: presence of brain metastases/unstable bone metastases; any conditions that would interfere with their ability to perform the exercises; syncope episodes within the past month; and participating in a structured exercise program at the time of consent. With a targeted sample size of 25 [26] for this feasibility study and assumed drop-out rate of 8–10% [27,28], we aimed to recruit 27 participants.

### 2.1. Intervention

The intervention was based on the Otago program [29], which is known for targeting strength and balance and designed for delivery three times per week [30]. The Otago program is effective in reducing falls and fall injuries in high-risk individuals and in increasing the proportion of older adults able to live independently in the community [31]. Specifically, the strength training component involved exercises targeting the lower body, such as calf raise, forward lunge, squat, abdominal plank, deadlift, donkey kick, and pockets. The balance component included static and dynamic exercises, including one-leg stand and various stages of tandem stand, progressing from holding onto furniture or objects to standing without support.

Each participant received a virtual orientation session by a Certified Exercise Physiologist (CEP), which included an overview of exercise safety precautions and emergency procedures; advice on home exercise set up (e.g., set up in a corner of a room or by a counter; choice in and placement of a sturdy chair; removal of rugs and other fall hazards); and orientation to the online exercise platform. In addition, participants attended a one-hour tutorial in small groups that reviewed how to perform their independent home exercises, which included explaining different levels of exercise and how to challenge their balance. Once participants learned the routine and techniques, they continued the regimen three times each week for 1 h for a total of 8 weeks. One session each week was hosted as a live group class facilitated on Zoom^®^ by one CEP, supported by a qualified exercise professional (QEP) and a practicum kinesiology student—one responsible for leading the exercises (QEP), one additional demonstrator (practicum student), and the other for safety monitoring (CEP). Participants were divided into two smaller groups with different session days scheduled (e.g., Monday/Wednesday and Tuesday/Thursday groups). During each virtual session, three different levels of exercise including seated options were provided to accommodate different levels of ability. The QEP demonstrated the standard and more difficult version and the practicum student demonstrated the easier seated version. The participants were also taught to ‘pin’ the video of the demonstrator for their recommended level of exercise. The second and third session of the week were carried out independently by each participant, with the support of tailored exercise programs created through the PhysioTec^®^ portal, which includes step-by-step instructions for each exercise maneuver, with videos to follow along. Each participant was provided with a personalized exercise log to record their exercise, keep track of progress, and stay motivated. Documentation of the individual exercise components included date and time, type and amount of exercise routines performed, time spent to complete the routines, perceived difficulty level, and any adverse events. The intervention is described in accordance with the Template for Intervention Description and Replication (TIDieR) checklist guide [32]. Participants were provided with contact information for the CEP for any follow-up questions.

### 2.2. Progression

Each participant’s exercise performance was closely monitored by the CEP. Exercise loads and progressions were titrated accordingly during each exercise session. Progression of balance training occurred by graduating from holding onto a stable structure, to performing the routine independent of support [30]. For strength training, each exercise progressed initially by repetitions from 10 to 15 for 2 sets and then by resistance level. When the resistance was increased, the number of repetitions was started again at 10. Resistance was increased in one of the following ways: (1) positioning—increasing resistance by progressing the exercise from gravity eliminated to gravity resisted, and, for example, by increasing lever length; (2) band tension—resistance was increased by shortening the length of the band (placing the hands further away from the ends of the band) such that the tension of the band was increased; (3) band strength—resistance was increased by progressing in band color (tension) or doubling of bands. Participants without equipment at home were provided with options for household items to use as resistance.

### 2.3. Safety Considerations

For safety purposes, we collected information on support persons in the home. An emergency contact, involving someone in the same residence or, for those living alone, a neighbor, was collected from each participant in the event of an emergency. Participants were not required to be accompanied during the session or assessments. Since the exercises and assessments were largely chair-based, or performed while holding onto or within reach of a sturdy surface, or on the floor in sitting/lying positions, the risk associated with falls was minimal.

### 2.4. Assessments and Outcome Measures

Outcomes were assessed virtually at baseline, 4 weeks (mid-intervention), and 8 weeks (end of intervention). The baseline assessment included a survey (sociodemographic information, fall history) and outcome measures. Functional status was also assessed using the 7-item, validated Older Americans’ Resources and Services (OARS) Instrumental Activities of Daily Living (IADL) scale [33]. End-of-study assessments included outcome measures and exit questions to collect participant feedback regarding the intervention for any difficulties related to adherence and for future improvement. All outcomes assessments involved two CEPs on different computers in different locations, neither of whom were involved in the analysis of study results. Prior to each assessment, participants were instructed in the set-up of their space. The tests required minimal materials and set-up, which involved a chair against the wall and a sturdy surface (e.g., kitchen counter). Participants were provided with instructions regarding the test and also opportunity to practice performing the test before they were assessed. In the event of video lag, the participant and/or their support person were instructed to also count repetitions. 

### 2.5. Feasibility

Feasibility was assessed using recruitment, retention, adherence, and outcome capture rates, along with acceptability [34]. Acceptability was assessed using exit questions, informed by previous research on exercise programs for older adults [35] and the expert consensus of the research team.

### 2.6. Lower Body Strength

The 5-times chair stand test was used to assess lower body strength, and it measures the time to complete five sets of sit-to-stand maneuvers from a standard chair. This common tool for measuring lower limb strength in older adults has an optimal cutoff of 10 s [36].

### 2.7. Balance

Static balance ability was measured using the 4-stage balance test in which participants were asked to maintain their balance for 10 s in each of the four progressively difficult positions (side-by-side, semi-tandem, full-tandem, and one-leg stand) [37]. The amount of time they succeed in maintaining the various positions was recorded based on the following criteria: side-by-side 10 s, semi-tandem 10 s (1 point); tandem 10 s (2 points), tandem 3–9.99 s (1 point) [38].

### 2.8. Falls

Falls (as outcome) were assessed at baseline and end-of-study by participant self-report following recommendations for fall assessment as outlined by the American Geriatrics Society/British Geriatrics Society (AGS/BGS) [39] Information regarding fear-of-falling (FOF) was collected using the Fall Efficacy Scale-International [40].

### 2.9. Statistical Analysis

Descriptive statistics (e.g., means, standard deviations, frequencies, percentages) were used to describe patient characteristics (i.e., age, gender, cancer site, treatment type) and functional status. 

#### 2.9.1. Feasibility

Descriptive statistics (numbers, proportions) were used to analyze recruitment rate, retention, adherence, outcome capture, completion, and quantitative data from exit questions. 

#### 2.9.2. Efficacy

Descriptive statistics (medians, numbers, proportions) were used to report chair stand time, balance scores, and fall rates at baseline and end-of-study to reflect absolute changes. Wilcoxon signed ranks were used to compare baseline and end-of-study measurements.

## 3. Results

Twenty-seven older adults with cancer (mean age 70.6, SD 4.6; 54% female) participated in this study. Four participants (15%) had at least one IADL impairment. The most common cancer sites were breast (46%) and prostate (38%). Seventeen (65%) were on active treatment, with 88% receiving hormones and 12% chemotherapy. One participant withdrew due to personal reasons unrelated to the program. The remaining 26 participants completed the intervention. Each exercise routine took approximately one hour to complete. Nearly 42% (*n* = 11) of participants perceived it was ‘a bit difficult’, followed by 31% (*n* = 8) who perceived it was ‘somewhat difficult’. No adverse events related to the exercise program were reported. Interference of video lag related to poor connection was rare and only occurred with two participants during assessments; in one case, the assessment was rescheduled. Descriptive results for participants who completed the training program are presented in Table 1.

### 3.1. Feasibility

Of the 45 patients expressing interest, 27 participated (recruitment rate 60%). Attendance rates for the virtual and independent components were 97.6% and 84.7%, respectively. Attrition and program completion rates were 4% and 96%, respectively. Outcome capture was 100%. Of note, some participants (*n* = 6) required help (by CEPs via phone call) with getting into the Zoom meeting for the first couple of exercise group classes. Participants perceived the program as rewarding and enjoyable (100%), felt it prepared them to exercise on their own (92%), were confident to continue exercising on their own (81%), and would recommend the program to others (100%). Participants seemed satisfied with the program. Supplementary Tables S1 and S2 provide details from the exit survey. 

### 3.2. Efficacy

Median balance scores at baseline and end-of-study were identical (4; IQR = 0). Median chair-stand time decreased from 9.2 s (IQR = 3.13) to 7.7 s (IQR = 4.6) over the course of the study; six participants (23%) surpassed the minimal clinically important difference (MCID) of 2.3 s [41]. A statistically significant improvement in lower body strength was detected post-intervention (*p* = 0.001, *r* = 0.68); no difference was detected in balance (*p* = 0.059, *r* = 0.37). Median FES-I scores increased from 19 (IQR = 2) to 21 (IQR = 6) between baseline and end of intervention. Forty-one percent of participants (*n* = 11) experienced an increase in fear-of-falling, whereas 11% (*n* = 3) experienced a decrease in FOF. At baseline, 23% (*n* = 6) had ≥2 falls over the past year. Two falls (unrelated to the exercise program) were reported during the 8-week intervention period (see Table 2).

## 4. Discussion

Findings show the hybrid, 8-week strength and balance training program was highly feasible among older adults with cancer, and it may have potential for improving lower body strength, which is an important index of falls. Despite a significant improvement in lower body strength at week 8, the majority of participants did not reach the MCID for the five-times chair stand, and no significant improvement was detected in balance. One possible explanation is that the majority of participants had good baseline lower body strength and balance to begin with. Hence, the benefits of this intervention may not be realized compared to those starting with greater frailty or weaker physical performance status. A small sample size also contributed to the inability to detect differences. We used static balance (quiet standing) as opposed to dynamic balance (maintain stability while executing a prescribed movement [42]), as in the Timed Up and Go test [43], because it was more feasible to assess virtually on camera. This may also have impacted our findings, as static balance is less challenging [44] and hence may be less sensitive to change. Research in the general geriatric population shows that combined resistance and balance training interventions are the most effective in reducing falls [45,46]. Additionally, exercise programs with a telepresence can have a positive impact on fall risk among older women [47]. Pre- and post-intervention fall rates were both low, but trending, suggesting the efficacy of the intervention. Our data suggest an increase in FOF among participants over the 8-week period. This may be in part due to increased awareness about falls and risk of falls by virtue of participating in this study. Moreover, all baseline testing was conducted in the early fall, whereas all participants finished the program in the winter months from late November to mid-December, which is a time when snow and ice are known to increase the likelihood of experiencing a fall. Other factors such as declining health status and cancer progression may have been at play; however, we were unable to access participants’ medical records to collect relevant information that could provide context. A unique feature of our study was the opportunity for participants to exercise in the comfort of their own homes while enjoying the advantage of exercising in a group setting with peers. The good adherence rate is in line with home-based exercise programs [48,49] and supervised exercise programs [50] in older adults with cancer. The convenience offered by our home-based program, along with the opportunity to exercise with peers, especially under the prevailing social restrictions, may have promoted adherence. Research shows supervised group sessions are welcomed by people with cancer [51], as they allow for a socially stimulating environment that promotes adherence and retention [52]. Evidence from the non-cancer setting shows that virtual gym offerings are highly usable and accepted, and the socialization aspect motivates older adults to adhere to the training program [31,53]. Shared identity and camaraderie are also highlighted as important in the older cancer population [54]. Social support, even in remote form, can be valuable for the maintenance of PA [55].

The pandemic has led to a decrease in PA in older adults [56,57], as well as an emphasis on the importance of older adults with cancer minimizing outings and observing physical distancing [24]. While an increasing proportion of older adults are being vaccinated, COVID infections due to new variants are rising, and the pandemic may continue into the foreseeable future. Exercising in facility-based programs, in particular those delivered in group format, may provide the social factors that improve long-term adherence and enhance the exercise experience for adults with cancer [58], but it may not be feasible given the ongoing pandemic and added vulnerabilities of older adults with cancer [24]. Thus, exercise programs must pivot from conventional clinic- and community-based models to programs that can be delivered in the home setting in a safe and reliable manner, ideally with monitoring by telehealth exercise oncology [59]. Older patients in the present study were able to handle the technology required to complete the virtual exercise program using an online conferencing platform. This is valuable information given the possibility of future pandemics, along with the increasing prevalence of e-health interventions and the delivery of exercise interventions via telehealth platforms (e.g., Zoom^®^, Skype^®^, MS Teams^®^, FaceTime^®^) for older adults with cancer [60]. Fall prevention interventions in the general geriatric population are increasingly using technology, especially e-interventions, to reduce falls in community-dwelling older adults, and have demonstrated success in this and in improving balance and strength [61]. It is important for people to exercise in a safe environment with the support of exercise specialists [59]. Having a cancer-specific experienced professional facilitator deliver and monitor the exercise sessions was an advantage of this home-based program, and it is seen as a facilitator in exercise program participation in the older adult population [53,62]. The emergence of innovative exercise platforms, such as virtual instructor-led sessions, are particularly useful in enabling older adults to exercise during this time, and it can capitalize on existing telemedicine platforms such as tablets and phones to support delivery [54].

### Strengths and Limitations

This study provides a much-needed initial assessment of the potential to deliver an exercise program to older adults with cancer by improving physical performance and reducing falls using a remote, hybrid approach. To the best of our knowledge, this is the first study specifically targeting strength and balance training to improve physical performance and prevent falls in older adults with cancer utilizing an e-intervention. Traveling to an exercise location weekly and performing exercise in a group environment may pose barriers to patients in terms of transportation, time, and inflexible program hours [63,64] and can bring undue risks for older adults with cancer, especially within the context of a global pandemic. A strength of this study was our ability to circumvent these issues while still allowing participants the opportunity to exercise in a group setting. The CEPs delivering the intervention had received prior cancer-specific training and had experience working with adults with cancer. Participants were able to see their peers during the session, thus ensuring a sense of community and camaraderie, which is important in the older cancer population [65,66,67]. The use of the Physiotec^®^ online platform for developing individualized regimens and titrated progressions and the monitoring of adherence by the CEP enabled participants to stay engaged. The positive survey feedback, along with the high adherence and completion rates, are evidence of the feasibility of this type of exercise program. No adverse events related to this exercise program were reported. The study is not without limitations. First, all participants who contacted us and expressed interest to participate owned a tablet device or computer. Therefore, the feasibility for those who do not own a device is not known, and the possibility of sampling bias cannot be excluded. Hence, future studies may also need to consider potentially incorporating administrative or technical support (e.g., to navigate device or online meeting platforms), as well as providing tablet devices, along with internet access and training support, to eliminate this barrier to entry. Second, the lack of a control group precludes a well-rounded understanding of how this intervention compares with standard care. Due to COVID-related research restrictions, we were unable to perform chart review at the cancer center to collect information such as cancer stage, treatment history, and comorbidity to provide information on participant characteristics. We were also unable to collect data that would allow us to explore how various factors including cognitive function, socioeconomic status, computer literacy, and other psychosocial factors could potentially impact older adults’ ability to participate in and complete the intervention. Additionally, the enrolling patients had all previously taken part in the ACE program, which may have led to selection bias. Although none of our participants had been formally exercising in the approximate 7-month period between the start of the pandemic and consenting to the study, this was a more self-motivated group who understood the benefits of staying physically active and, hence, may not be representative of the older cancer population. However, all participants were over the age of 65 and none had taken part in a virtual exercise program. Moreover, as the in-person ACE program is offered in 17 locations, participants were not necessarily familiar with the study team. As our intent was to test the feasibility of an intervention protocol to address falls, using a group already familiar with exercise removed some of our initial safety concerns around the virtual delivery and allowed us to test the delivery of the protocol in a virtual environment. Additionally, the heterogeneity in terms of cancer site, stage, treatment modality, especially given the small sample size, also precludes the generalizability of this study. Finally, study results should be interpreted in light of the small sample size, the low risk group for falls, and the short intervention and follow-up period. However, the remit of this study was mainly to assess the feasibility of the intervention protocol and virtual delivery method. The estimates for effect sizes in this study may have utility in informing future trials.

## 5. Conclusions

This study addresses an important gap in knowledge in geriatric oncology by providing data regarding the feasibility and potential effectiveness of a remotely delivered e-intervention exercise program targeting physical performance and falls in older adults with cancer. We found this program to be highly feasible. A larger study, in the form of a randomized controlled trial, is needed to explore future clinical outcomes.

## Figures and Tables

**Table 1:** Participant characteristics (*n* = 26).

Characteristics	% (*n*)
**Age**	
Mean ± SD	70.6 ± 4.6
Range	65–76
**Female sex, % (*n*)**	54% (14)
**Type of Cancer**	
Breast	46% (12)
Prostate	38% (10)
Colorectal	15% (4)
**On active cancer treatment % (*n*)**	65% (17)
Receiving hormonal therapy	88% (15)
Receiving chemotherapy	12% (2)
**Treatment completed in the past**	
Radiation	81% (21)
Surgery	77% (20)
Chemotherapy	54% (14)
Hormone	42% (11)
Biological therapy	4% (1)
**Have at least 1 IADL limitation**	15% (4)
**High fear of falling ^**	19% (5)

IADL—instrumental activities of daily living, ^ Based on Fall Efficacy Scale—International cutoff of 23.

**Table 2:** Chair stand time, balance score, and fall rate at baseline and end of intervention.

Assessment	Baseline	Mid-Intervention	End of Intervention	Significance
Five times chair stand time, median (IQR)	9.2 (3.13)	7.75 (4.4)	7.7 (4.6)	*p* = 0.001 (*r* = 0.68)
Balance score, median (IQR)	4 (0)	4 (0)	4 (0)	*p* = 0.059, (*r* = 0.37)
Number of participants who had ≥2 falls in past year	6	N/A	N/A *	N/A
Fall efficacy score **, median (IQR)	19 (2)	N/A	21 (6)	*p* = 0.0005

* Two participants reported one fall at 8 weeks (end of intervention), ** based on a possible score range of 16–64.

## Data Availability

The data presented in this study are available on request from the corresponding author. The data are not publicly available due to privacy reasons.

## Supplementary Materials

The following are available online at https://www.mdpi.com/article/10.3390/curroncol28060374/s1, Table S1: Findings from exit survey, Table S2: Free-text comments from exit survey.
