# Supplementary material for: Feasibility of a Remotely Delivered Strength and Balance Training Program for Older Adults with Cancer
Source: Curr Oncol. 2021 Nov 2;28(6):4408–19. doi: 10.3390/curroncol28060374 (PMC8628693; doi:10.3390/curroncol28060374)
Supplement: Supplementary file 1 [file curroncol-28-00374-s001.zip › Supplementary Table S1_Findings from exit survey.pdf]

Supplementary Table S1 – Findings from exit survey

Survey Item *	Participant Response % (n)
The stable program improved my leg strength	62% (16) answered quite a bit or very much
The stable program improved my balance	65% (17) answered quite a bit or very much
The program staff were knowledgeable	100% (26) answered quite a bit or very much
The program staff were supportive	100% (26) answered quite a bit or very much
Service received from program staff	100% (26) answered above average or excellent
How enjoyable was the exercise program?	100% (26) answered quite a bit or very much
How beneficial do you think it will be for you to continue exercising?	100% (26) answered quite a bit or very much
How motivated are you to continue to exercise?	92% (24) answered quite a bit or very much
How confident are you to continue exercising on your own?	81% (21) answered quite a bit or very much
Do you feel that the STABLE program prepared you to exercise on your own?	92% (24) answered quite a bit or very much
It was rewarding	100% (26) answered quite a bit or very much
It was a waste of my time	100% (26) answered not at all
It will be useful for research helping others	88% (23) answered quite a bit or very much
It was useful to me personally	100% (26) answered quite a bit or very much
I would recommend the exercise program to other cancer patients	100% (26) answered quite a bit or very much

* On a Likert scale, participants were to select from a list ranging from: not at all, a little bit, somewhat, quite a bit, or very much
