# Supplementary material for: Feasibility of a Remotely Delivered Strength and Balance Training Program for Older Adults with Cancer
Source: Curr Oncol. 2021 Nov 2;28(6):4408–19. doi: 10.3390/curroncol28060374 (PMC8628693; doi:10.3390/curroncol28060374)
Supplement: Supplementary file 1 [file curroncol-28-00374-s001.zip › Supplementary Table S2_Free text comments from exit survey.pdf]

Supplementary Table S2 – Optional free text comments in exit survey

“It is a bit of a let down when the programme finishes. I can't help wondering if there isn't somewhere I could continue on with something similar, even at a cost. I think I am much more likely to continue exercising when I'm involved in a formal setting such as this. The once a week zoom plus specified home exercises in between worked very well to keep me at it. On my own my motivation tends to disappear very quickly despite being well aware of the benefits!”
“The STABLE program was extremely well run. The Home exercises were explained in detail for the different levels that was so beneficial. The 2 facilitators did an excellent job. A great program.”
“A sincere thank you to <<the CEPS>> for their patience with us all. They were caring and knowledgeable.”
“The program was very well planned, providing knowledge and purpose to us. Staff were more than accommodating, answering our specific questions and keeping us in good humour!! Music was great.”
“Great program. I hope that it will continue.”
“You should get the information we put on the sheet after each home self-administered session. Otherwise well done!!”
“……(the CEPs) did an excellent job. Zoom worked so well.”
“I loved the program. Doing the exercises at home with professional supervision via Zoom is outstanding and comfortable. Personally, I would appreciate the preparation of a general video with warm-up exercises that will prepare participant to initiate their regular workout or class. Thank you. The team leading the program were very encouraging. It was always enjoyable to be exercising with them. Having the demonstrations of the exercises was helpful. It's not always easy to know how to do an exercise properly. The team was able to provide alternatives to accommodate some personal health issues so that I could still do the exercises. That was very important to me. I, personally, do much better at following an exercise program when I have someone to share it with, so this opportunity was wonderful. Although I would rather be in a gym with friends, this worked very well fo(r) me. Thanks to the whole team for leading some great exercise sessions.”
“Stable program is excellent for balance and confidence building. Some of the Cancer drugs actually have a VERY negative effect on muscle regeneration and rebuilding as such, recovery times are greatly increased from normal. Extended recovery times produce muscle pain and discomfort that may not be tolerable by some participants.”
“I feel this teaching is extremely important to have a healthy lifestyle and it should be part of any wellness after health issues.”
“The staff was very accommodating to our levels of needs and ability. Excellent staff support!”

Note: Thematic analysis was not performed due to the small number of free-text responses collected from the exit survey.
